# Comparative Prognostic Value of Glomerular Filtration Rate, Serum Cystatin C, Beta‐2‐Microglobulin and Albuminuria for Death and Chronic Kidney Disease Progression

**DOI:** 10.1002/jcla.25139

**Published:** 2024-12-23

**Authors:** Isis Cerezo, Barbara Cancho, Jorge Alberto Rodriguez Sabillon, Alberto Jorge, Alvaro Alvarez Lopez, Julian Valladares, Juan Lopez Gomez, Jorge Romero, Nicolas Roberto Robles

**Affiliations:** ^1^ Servicio de Nefrologia Hospital Universitario de Badajoz, Universidad de Extremadura Badajoz Spain; ^2^ Servicio de Bioquímica Clínica Hospital Universitario de Badajoz, Universidad de Extremadura Badajoz Spain; ^3^ Servicio de Medicina Interna Hospital Universitario de Badajoz, Universidad de Extremadura Badajoz Spain

**Keywords:** albuminuria, beta‐2‐microglobulin, chronic kidney disease, cystatin C, glomerular filtration rate, mortality

## Abstract

**Aims:**

Serum creatinine and albuminuria are the core of most CKD prediction and progression risk models. Several biomarkers have been introduced to improve these results such as beta‐2‐microglobulin (B2M) and cystatin C (CysC). Nevertheless, few clinical comparisons of these biomarkers are available. We have compared serum B2M levels with albuminuria, CysC levels, and the CKD‐EPI GFR equations.

**Designs and Methods:**

A sample of 434 patients were studied: 234 males and 200 females, the mean age was 58.3 ± 15.0 years, and 33.4% have diabetes mellitus. In all patients, plasma B2M, CysC, creatinine, and urinary albumin excretion were analyzed. EGFR was calculated using CKD‐EPI equations for creatinine, CysC, and creatinine‐CysC. The risk of death and CKD progression was evaluated using ROC curves and Cox proportional hazards survivorship models.

**Results:**

For mortality, the highest area under the curve (AUC) was for CysC (0.775, 0.676–0.875). The lowest sensitivity was shown by eGFR (creatinine) (0.298, 0.195–0.401, *p* < 0.001), eGFR (CysC) (0.216, 0.118–0.314, *p* < 0.001), and eGFR (creatinine + CysS) (0.218, 0.124–0.312, *p* < 0.001). For progression to advanced CKD, the highest AUC was for CysC (0.908, 0.862–0.954). The lowest sensitivity was shown by eGFR (creatinine) (0.184, 0.106–0.261, *p* < 0.001), eGFR (CysC) (0.095, 0.048–0.14, *p* < 0.001), and eGFR (creatinine+ CysC) (0.087, 0.040–0.134, *p* < 0.001). CysC, after age, was the second‐best marker of life risk. Contrariwise, for CKD progression, CysC, and albuminuria were the best markers.

**Conclusions:**

The best biomarker of mortality and risk of progression to CKD was CysC. Albuminuria and B2M were the next best options to be used. The lowest sensitivity was shown by estimated eGFR.

## Graphical abstract

Serum creatinine and albuminuria are the core of most CKD prediction and progression risk models. We have compared the prognostic powers of serum B2M levels with albuminuria, CysC levels, and the CKD‐EPI (CKD‐Epidemiology Collaboration) GFR equations for mortality and CKD progression. The best biomarker of mortality and risk of progression to CKD was CysC. B2M and albuminuria were the next best options to be used. The caption is comparative statistical significances for every AUC.

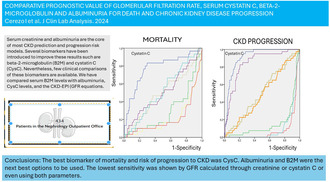

## 1 Introduction

Classically, serum creatinine and albuminuria are the core of most CKD prediction and progression risk models. However, both biomarkers only show alterations relatively late in the disease progression and thus are not suitable for early CKD diagnosis [1]. Several biomarkers have been introduced to evaluate the renal function. Serum creatinine (SCr) measured by a blood test can inform the physician how well the kidneys are working. When the kidneys are not working well, the SCr concentration increases. Although SCr can be used to clinically interpret SCr, nephrologists prefer using estimated glomerular filtration rate (eGFR) obtained from GFR equations and expressed in mL/min/1.73 m^2^ because it better reflects the inverse association between eGFR and creatinine. It is considered the principal measure of kidney function and, together with albuminuria, is a relevant prognostic factor for the development of end‐stage kidney disease. Due to the strong association between estimated eGFR and clinical events, such as commencement of dialysis, cardiovascular outcomes, and all‐cause death, eGFR is crucial for clinical decision‐making in terms of scheduling follow‐up and pharmacological interventions and planning renal replacement therapies in advanced chronic kidney disease. In this regard, according to the KDIGO (Kidney Disease Improving Global Outcomes) guidelines, the patients’ risk is classified on the basis of those parameters, eGFR and SCr [2]. In the same way, the most used equation for calculating the prognosis of patients uses the same parameters [3].

CysC, a low molecular‐weight protein freely filtered by the kidneys, is an established alternative to creatinine for GFR estimation. Unlike creatinine, CysC is fully reabsorbed and catabolized in the proximal renal tubules, with no tubular secretion, in healthy subjects (Table 1). Most recent recommendations on renal function evaluation suggest that the better option is to estimate GFR simultaneously from SCr and CysC. The last biomarker also has a good relationship with life survival and CKD progression [4].

Albuminuria has been recognized for a long time as a hallmark of diabetic nephropathy and a predictor of cardiovascular events in people with diabetes mellitus [5, 6]. However, recent evidence indicates that increases in urinary albumin excretion even within the normal range, and thus below the specific cutoff for the definition of microalbuminuria, may predict renal and cardiovascular risk in adults with chronic kidney disease as well as in the general population [7, 8].

Like CysC, beta‐2‐microglobulin (B2M) is a low molecular weight protein that is filtered by the glomeruli and degraded by the tubules [9]. Like the previous biomarkers, B2M has been shown to be useful in estimating GFR; is less influenced by age, sex, and race than creatinine; and is more strongly associated with death and cardiovascular disease compared to creatinine or estimated GFR [10].

The aim of this study was to find a better renal prognostic biomarker. In this regard, we compared serum B2M levels with albuminuria, CysC levels, the CKD‐EPI (CKD Epidemiology Collaboration) creatinine‐based equations, and the creatinine‐CysC combined equation in these patients looking the prognostic power for death and CKD progression.

## 2 Designs and Methods

A sample of 434 patients was studied: 234 males and 200 females; mean age was 58.3 ± 15.0 years; of them, 33.4% had diabetes mellitus, 93.0% had high blood pressure (98.1% treated with ACE inhibitors or angiotensin receptors blockers), 48.0% had dyslipidemia, 11.3% were diagnosed of malignancy, and 6.2% have thyroid disease. The patients were consecutively recruited in the Nephrology outpatient's clinics of our hospital, an academic institution with the highest specialization and regional coverage. The causes of CKD are summarized in Table 2. Follow‐up visits were programmed according to KDIGO recommendations for the different levels of renal disease. Our hospital uses electronic clinical reports; therefore, even when the patient is lost of direct follow‐up, we can search for life and analytical status. Table 1 shows the causes of follow up.

**TABLE 1:** Causes of follow‐up in the outpatient's office.

	All	Men	Women
Glomerulonephritides	80	47	33
Diabetic nephropathy	28	15	13
Nephrosclerosis	49	36	13
Chronic pielonephritis	33	20	13
Interstitial nephritis	52	28	24
High blood pressure	123	54	69
Autosomic‐dominant policystic disease	13	2	11
Lupus nephritis	12	1	11
Nephrectomy	24	15	9
Other/unknown	20	16	4
	434	234	200

**TABLE 2:** Anthropometric data.

	Global sample	Dead	Renal replacement therapy
*n*	434	68	46
AGE (years)	58.3 ± 15.0	57.8 ± 15.9	58.0 ± 13.3
GENDER (Male/Female) %	53.9/46.1	52.9/47.1	50.0/50.0
IMC	29.7 ± 6.2	30.5 ± 7.4	30.5 ± 6.0
DIABETES MELLITUS	33.4%	38.2%	39.1%
HIGH BLOOD PRESSURE	93.0%	97.3%	96.9%
DYSLIPEMIA	48.0%	50.3%	49.3%

Serum CysC was measured using a BNII nephelometer (Dade Behring Inc., Deerfield, IL, USA) that used a particle‐enhanced immunonephelometric assay (N Latex Cystatin‐C). The assay range is 0.195–7.330 mg/L, with the reference range for young healthy individuals reported as 0.53–0.95 mg/L. The cut point for the highest quartile of serum cystatin distribution was 1.05 mg/L, and this was the limit used to define high plasma cystatin levels. Albuminuria was measured in 24‐h urine collection using an immunonephelometric assay on the same device. The protein measurement procedure in urine was the method of pyrogallol red‐molybdate using the Advia 2400 Chemistry System Analyzer (Siemens Health Care Diagnosis, Deerfield, IL, USA). GFR was estimated from serum creatinine measured through the standardized Jaffé method (IDMS) using the CKD‐EPI equation [11, 12]. The GFR was also estimated from creatinine and CysC using the CKD‐EPI for creatinine‐CysC [13]. Serum B2M was measured using a Cobas 6000 device (Roche Diagnostics, Indianapolis, IN, USA) that measured urinary B2MG immunoturbidimetrically.

Patients were classified according to K/DIGO stages of chronic renal disease using the CKD‐EPI formulation for creatinine: 13.6% were in stage IV or V, 30.9% were in stage III, and the remaining patients had GFR higher than 60 mL/min (39.1%).

Microalbuminuria was defined as a urinary albumin excretion > 30 and < 300 mg/day; macroalbuminuria was diagnosed when albuminuria was equal to or higher than 300 mg/day. CKD progression was defined as reaching KDIGO stage 5 or starting renal replacement therapy.

All procedures performed in studies involving human participants were in accordance with the ethical standards of the institutional and/or national research committee and with the 1964 Helsinki Declaration and its later amendments or comparable ethical standards. The ethical approval was supplied by the University of Extremadura Ethics Committee (#21/2014). Informed consent was obtained from all individual participants included in the study.

### 2.1 Statistics

Results are expressed as mean ± 1 SD. All statistical tests were two‐sided. *p* values lower than 0.05 were considered significant. Associations between the different biomarkers and risk of death or reaching renal replacement therapy were assessed using Cox proportional hazards survivorship model. Hazard ratios (HR) and corresponding 95% confidence intervals (CI) were calculated, and a *p* value of < 0.05 was considered to be statistically significant. All variables achieving a significance level of *p* < 0.1 in univariate analysis were considered for inclusion in the construction of the Cox model. The predictive value of the biomarker regarding the risk of death and CKD progression was evaluated with ROC curves. The DeLong test was used to detect differences between the area under the curve (AUC) of these models. The data were analyzed using the IBM statistical program SPSS Statistics V.21 (IBM Corporation, Armonk, NY, USA). Additional Cox regression procedures were carried out to control for classic cardiovascular risk factors.

## 3 Results

From the global sample, 46 subjects reached the KDIGO stage 5 and 68 participants died (19 after reaching the KDIGO stage 5). Anthropometric data are shown in Table 2.

**FIGURE 1:**
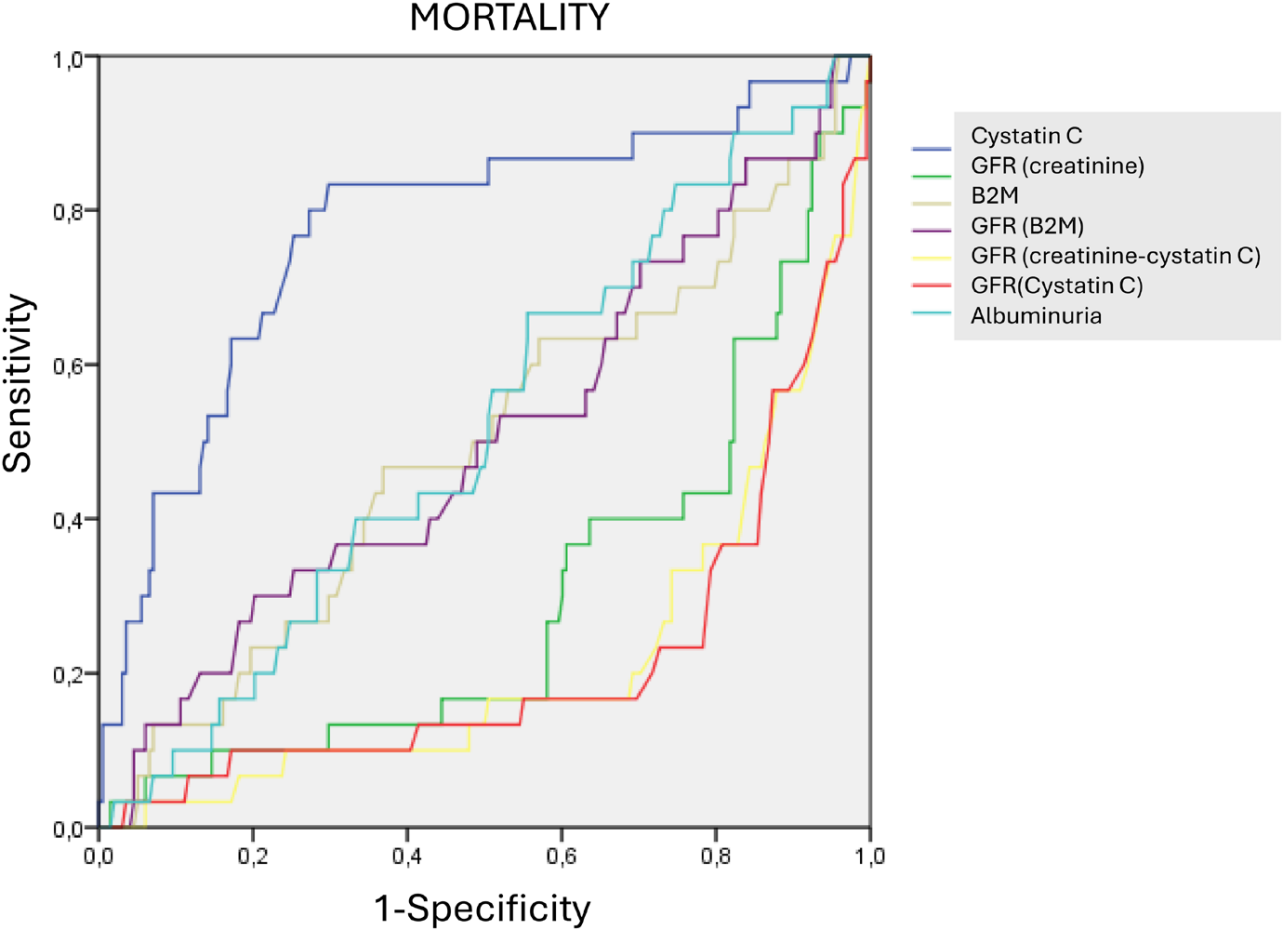
Comparison of ROC curves for mortality prognosis.

For risk of mortality, the highest area under the curve (AUC) was for CysC (0.775, 0.676–0.875). Albuminuria (0.519, 0.413–0.615, *p* < 0.001 vs. CysC), B2M (0.496, 0.379–0.612, *p* < 0.001 vs. CysC), and eGFR (B2M) (0.505, 0.388–0.621, *p* < 0.001 vs. CysC) showed very similar values. The lowest sensitivity was shown by eGFR (creatinine) (0.298, 0.195–0.401, *p* < 0.001 vs. CysC), eGFR (cystatin) (0.216, 0.118–0.314, *p* < 0.001 vs. CysC), and eGFR (creatinine + cystatin) (0.218, 0.124–0.312, *p* < 0.001 vs. CysC) (see Figure 1).

**FIGURE 2:**
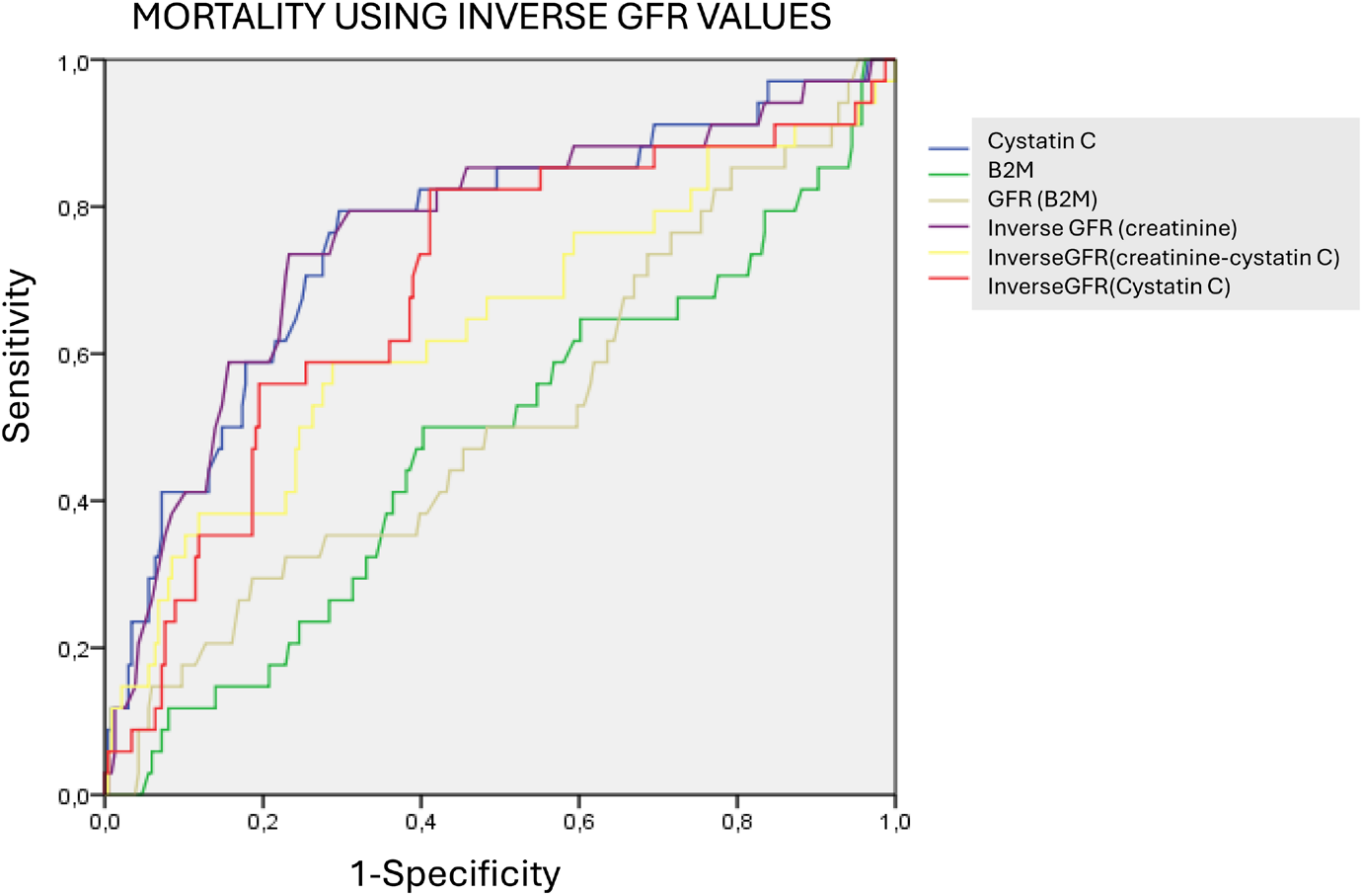
Comparison of ROC curves for mortality prognosis using inverse GFR values.

Nevertheless, if we calculate the inverse of eGFR from any one of the biomarkers, the results were more closely related to serum cystatin levels, but only eGFR (cystatin) reached the same sensitivity (see Figure 2): inverse of eGFR (creatinine) 0.578 (0.486–0.669, *p* < 0.001 vs. CysC); inverse of eGFR (creatinine+ cystatin) 0.667 (0.578–0.775, *p* = 0.027 vs. CysC); and inverse of eGFR (cystatin) 0.740 (0.663–0.816, *p* = 0.26 vs. CysC).

**FIGURE 3:**
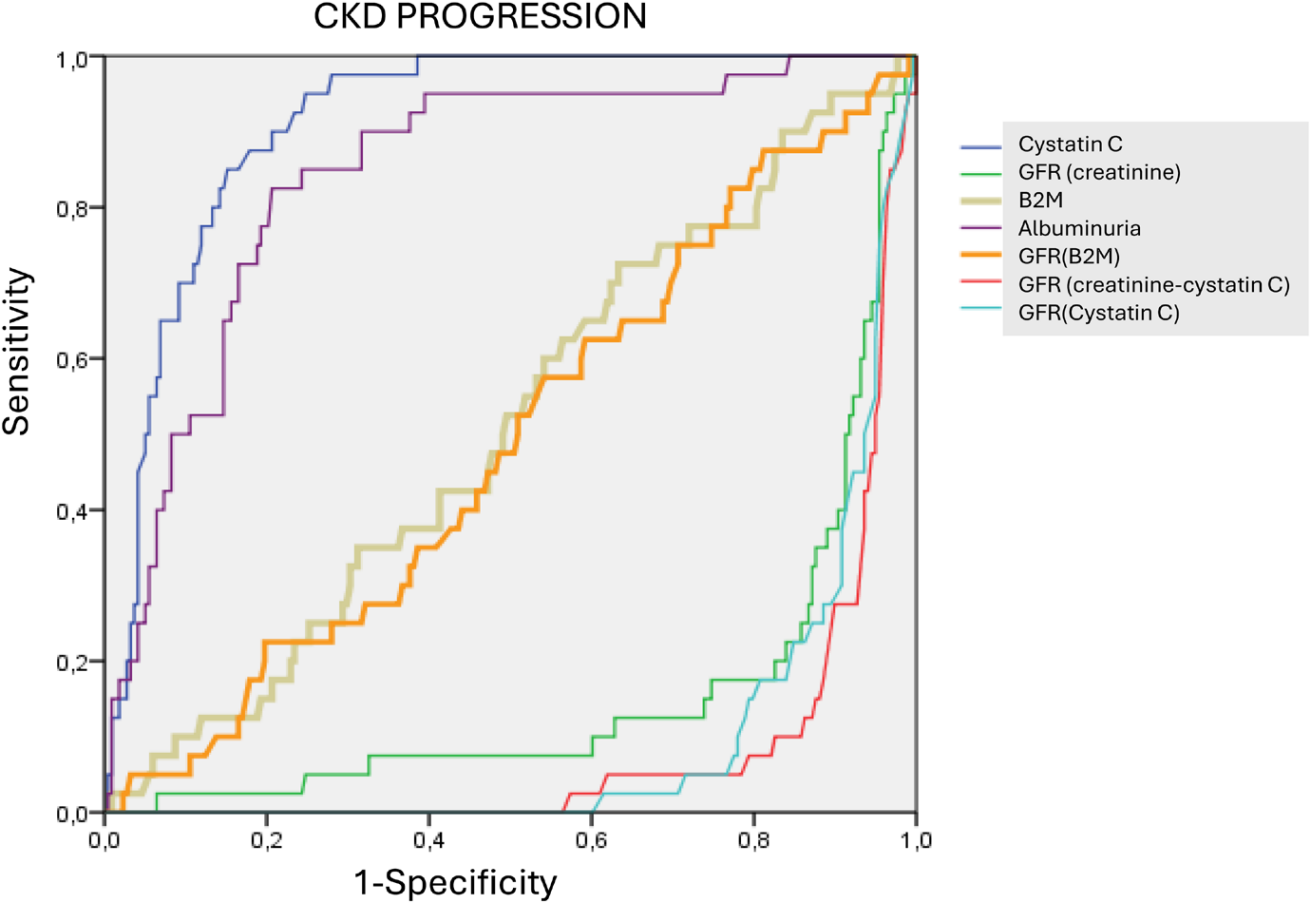
Comparison of ROC curves for CKD progression prognosis.

For progression to advanced CKD, the values were very similar but, again, the highest AUC was for CysC (0.908, 0.862–0.954). Albuminuria (0.514, 0.423–0.604, *p* < 0.001 vs. CysC), B2M (0.532, 0.432–0.632, *p* < 0.001 vs. CysC), and eGFR (B2M) (0.468, 0.368–0.568, *p* < 0.001 vs. CysC) showed very similar values. The lowest sensitivity was shown by eGFR (creatinine) (0.184, 0.106–0.261, *p* < 0.001 vs. CysC), eGFR (cystatin) (0.095, 0.048–0.14, *p* < 0.001 vs. CysC 2), and eGFR (creatinine +cystatin) (0.087, 0.040–0.134, *p* < 0.001 vs. CysC) (see Figure 3).

**FIGURE 4:**
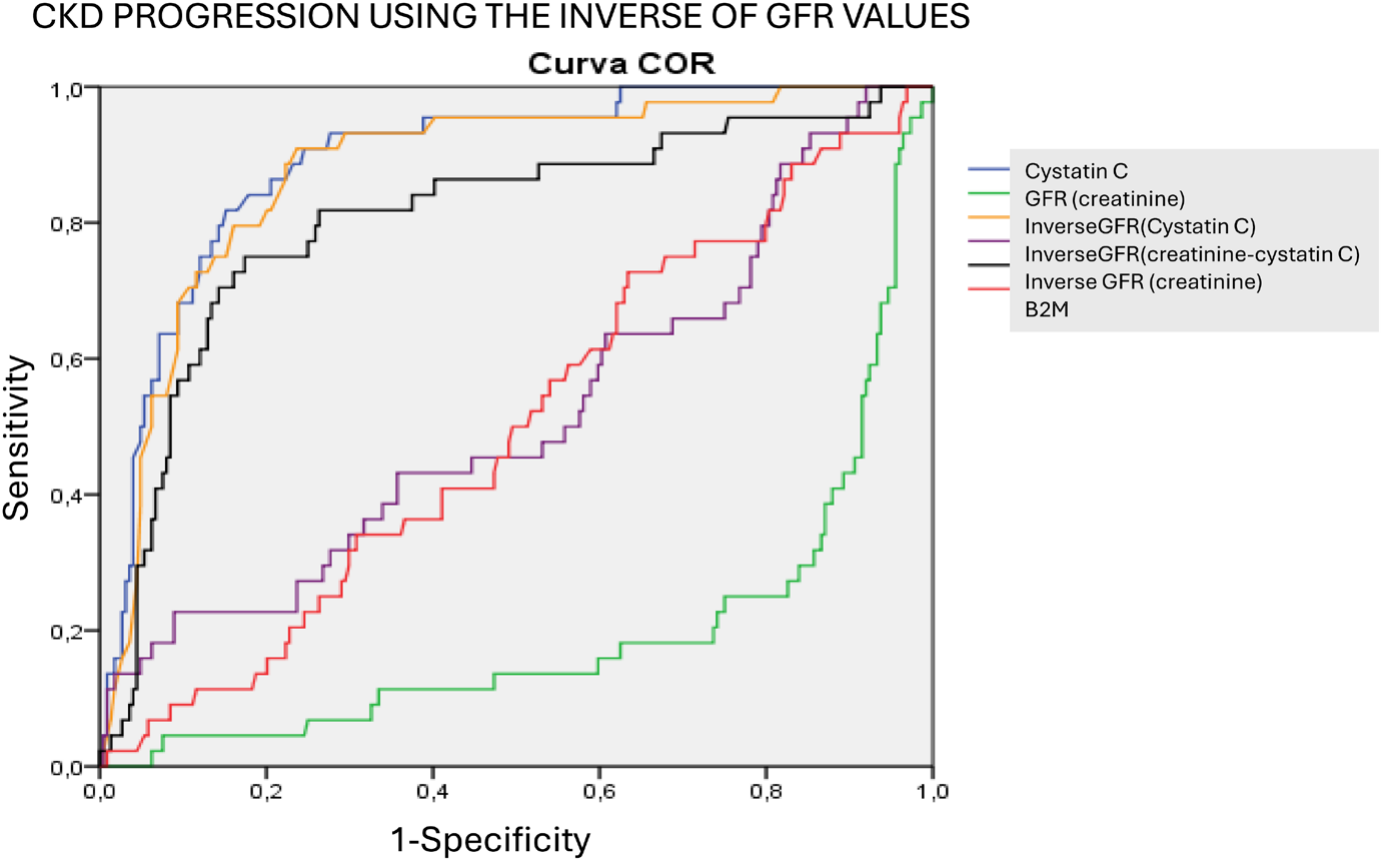
Comparison of ROC curves for CKD progression prognosis using inverse GFR values.

As for mortality, when evaluated with the use of the inverse of eGFR from any one of the biomarkers, the results were more closely related to serum cystatin levels but, again, no one of them reached the same sensitivity (see Figure 4): inverse of eGFR (creatinine) 0.748 (0.672–0.823, *p* < 0.001 vs. CysC), inverse of eGFR (creatinine+ cystatin) 0.566 (0.484–0.647), and inverse of eGFR (cystatin) 0.885 (0.844–0.926, *p* = 0.17 vs. CysC). All significances are shown in Table 3.

**TABLE 3:** Comparative statistical significances for every AUC.

	AUC	Cystatin C	B2M	GFR B2M	Albuminuria	GFR creatinine	GFR cystatin C	GFR creatinine‐cystatin C
Mortality								
Cystatin C	0.775		< 0.001	< 0.001	< 0.001	< 0.001	< 0.001	< 0.001
B2M	0.496	< 0.001		0.68	0.87	< 0.001	< 0.001	< 0.001
GFR B2M	0.518	< 0.001			0.81	< 0.001	< 0.001	< 0.001
Albuminuria	0.505	< 0.001				< 0.001	< 0.001	< 0.001
GFR creatinine	0.298	< 0.001					0.022	0.039
GFR cystatin C	0.218	< 0.001						0.96
GFR creatinine‐cystatine	0.216	< 0.001						
1/GFR creatinine	0.567	< 0.001	0.19	0.37	0.25	< 0.001	< 0.001	< 0.001
1/GFR cystatin C	0.740	0.26	< 0.001	< 0.001	< 0.001	< 0.001	< 0.001	< 0.001
1/GFR creatinine‐cystatine	0.667	0.027	0.002	0.006	0.003	< 0.001	< 0.001	< 0.001
CKD progression								
Cystatin C	0.908		< 0.001	< 0.001	< 0.001	< 0.001	< 0.001	< 0.001
B2M	0.532	< 0.001		0.23	0.74	< 0.001	< 0.001	< 0.001
GFR B2M	0.468	< 0.001			0.60	< 0.001	< 0.001	< 0.001
Albuminuria	0.514	< 0.001				< 0.001	< 0.001	< 0.001
GFR creatinine	0.084	< 0.001					0.012	< 0.001
GFR cystatin C	0.095	< 0.001						0.70
GFR creatinine‐cystatine	0.087	< 0.001						
1/GFR creatinine	0.748	< 0.001	< 0.001	< 0.001	< 0.001	< 0.001	< 0.001	< 0.001
1/GFR cystatin C	0.855	0.17	< 0.001	< 0.001	< 0.001	< 0.001	< 0.001	< 0.001
1/GFR Creatinine‐cystatine	0.566	< 0.001	0.53	0.069	0.34	< 0.001	< 0.001	< 0.001

Abbreviation: AUC, area under the curve.

Cox regression results for mortality risk are shown in Table 4. CysC, after age, was the second‐best marker of life risk in all models. Contrariwise, for CKD progression to end‐stage renal disease or KDIGO stage 5, CysC and albuminuria were the best markers, followed by eGFR (creatinine) (see Table 5).

**TABLE 4:** Cox regression for mortality risk.

	*B*	ExpB	95% CI	Significance
Age	0.076	1.079	1.037–1.122	0.000
Gender	0.657	1.930	0.897–4.150	0.093
Diabetes mellitus	0.601	1.823	0.869–3.824	0.112
Cystatin C	0.999	2.716	1.500–4.918	0.001
Albuminuria	0.000	1.000	0.999–1.000	0.422
GRF (Creatinine)	0.005	1.005	0.990–1.020	0.539
B2M	0.014	1.014	0.888–1.157	0.839

**TABLE 5:** Cox regression for chronic kidney disease progression risk.

	*B*	ExpB	95% CI	Significance
Age	−0.024	0.976	0.949–1.004	0.091
Gender	−0.341	0.711	0.326–6.814	0.392
Diabetes mellitus	0.789	2.202	1.078–1.001	0.030
Cystatin C	1.308	3.700	2.009–4.500	0.000
Albuminuria	0.001	1.001	1.000–1.552	0.000
GRF (creatinine)	−0.023	0.978	0.958–0.997	0.024
B2M	0.022	1.022	0.907–1.152	0.718

## 4 Discussion

In a sample of patients coming from the Nephrology outpatient clinic CysC was, by far, the best biomarker for death and progression to end‐stage kidney disease. In the Cox regression model, CysC again performed better than beta‐2‐microglobulin, GFR, and proteinuria to get a statistical association with those parameters.

CysC is a low molecular weight protein which has been proposed as a marker of renal function that could replace creatinine. Its concentration is mainly determined by glomerular filtration and is particularly of interest in clinical settings where the relationship between creatinine production and muscle mass impairs the clinical performance of creatinine. In the last years, a number of studies have evaluated its potential use in measuring renal function in various populations, but other potential uses in clinical settings have been proposed. For instance, Windhausen et al. [14] demonstrated that high CysC concentrations are associated with an increased risk of death and myocardial infarction in patients with non‐ST elevation acute coronary syndrome and elevated cardiac troponin T level, but this effect seems not to be associated only with renal dysfunction [15]. In this regard, multiple large‐scale observational studies and a meta‐analysis including participants with and without CKD have shown that CysC has closer, more linear associations with mortality and cardiovascular risk than serum creatinine levels [16]. Our results confirm this straight relationship and show again the usefulness of using CysC as a biomarker not only in CKD patients but also in general population with associated cardiovascular risk.

There are biological and clinical aspects that limit the use of cystatin C, such as obesity, thyroid dysfunction, systemic inflammation, and corticosteroid treatment [17]. There is conflicting evidence regarding the possible effect of diabetes mellitus on serum cystatin C values. Although positively related to an increased prevalence of diabetes in middle‐aged and older adults, Magnusson et al. [18, 19] found an association of serum cystatin C with metabolic syndrome but not with type 2 diabetes. However, causal associations with adverse outcomes could not be demonstrated in genome‐wide association studies, indicating that elevated serum cystatin C concentrations primarily reflect renal impairment [20].

B2M is a low molecular weight protein which is filtered by the glomeruli and almost completely reabsorbed by the proximal tubules. As its urinary excretion is residual, the increase in this protein has been proposed as a potential serum marker of decreased eGFR and also as a marker of tubular damage if its urinary excretion is elevated [21]. Moreover, B2M is not affected by ethnicity and is less influenced by age and gender than serum creatinine. It can also be evaluated in urine B2M alone or as a B2M‐to‐creatinine ratio [22]. Recently, it has been recommended a system of detailed examinations, including urinary B2M testing and ultrasonography, to detect congenital anomalies of the kidney and urinary tract, the most common, underlying disease in kidney failure with replacement therapy, which is often overlooked until the symptoms have become grave [23]. The associations between higher serum B2M and higher all‐cause mortality have been reported in both nondialyzed CKD [24, 25, 26] and hemodialysis patients [27]. Although we have found that plasma levels of B2M showed a fair relationship between patients mortality and the risk of CKD progression, this was less closer than the correlation showed by CysC. A GFR estimating equation using B2M levels has been developed [28] but has found no better results when comparing with plasma CysC alone, although it performed better than eGFR (creatinine), the most used biomarker all over the world.

The Multiple Risk Factor Intervention Trial (MRFIT) has followed a cohort of individuals for a long time. In middle‐aged men who are at above‐average risk for cardiovascular disease and did not have significant kidney disease, trace amount of protein on a casual urine dipstick, present in only a small percentage of men, was an important predictor of the 25‐year incidence of end‐stage renal disease [29]. In the same way, clinical trials consistently showed renoprotective effects of proteinuria reduction and led to the recognition that the antiproteinuric treatment is instrumental to maximize renoprotection [30, 31]. The MDRD study revealed a tight association between the reduction of albuminuria and the rate of GFR decline [32]. Studies in nonhuman models of CKD have suggested that albuminuria is an independent risk factor for the progression of renal disease [33]. It has been suggested that in progressive CKD, severe dysfunction of the glomerular capillary barrier to circulating proteins causes protein overload of tubular epithelial cells and intrarenal activation of complement that is responsible for the spreading of tubulointerstitial injury. The toxicity of albumin seems to be mediated by its initial endocytic uptake, although the importance of albumin itself versus protein‐bound molecules in the induction of irreversible tubular damage is not clear. Other proteins, including ultrafiltered transferrin and Ig, and the intrarenal complement pathway could play a predominant role [34]. Whatever the pathogenic mechanism involved, we found that proteinuria is a good biomarker for renal disease progression and mortality (very close to B2M), but CysC gets better results.

In recent years, there has been growing interest in the association between the severity of CKD and the risk of adverse outcomes such as mortality, cardiovascular events, and CKD progression. Several studies have reported strong associations between change in eGFR over 1 year and risk of end‐stage renal disease, [35, 36] major cardiovascular events [37], and mortality [38, 39] among CKD patients. Perkins et al. [40], for instance, examined the effect of the rate of eGFR decline on the survival of 15,465 NDD‐CKD patients receiving primary care at a single institution and reported 84% and 42% increases in mortality for those with declining (−4.8 mL/min/1.73 m^2^/year) and increasing eGFR (3.5 mL/min/1.73 m^2^/year), respectively, compared with those with stable eGFR. These studies have consistently demonstrated that 1‐year change in eGFR is strongly related to the risks of these outcomes, as this was the parameter used. This could explain why we have not found a close relationship between eGFR and mortality (or CKD progression) compared to CysC, B2M, or albuminuria because we have used a sectional approach classifying the patients by the first known value.

### 4.1 Strengths and Limitations

The main limitation of this study is the relatively small sample size. Nevertheless, although the use of CysC is growing in the clinical ground, this type of measurement is rare in clinical practice and, therefore, the resulting information for our results takes on greater importance. On the other hand, there is cumulated evidence of the predictive value of CysC on renal disease and general and cardiovascular mortality. Shlipak et al. [41], using data from the Cardiovascular Health Study found that CysC concentrations, showed strong associations with death, cardiovascular death, and major cardiovascular events among these participants. Serum creatinine concentrations had much weaker associations with each outcome and only predicted cardiovascular death. This study did not measure albuminuria, but some comparisons, showing again the higher predictive value of CysC are available [15].

### 4.2 Conclusions

The best biomarker of mortality and risk of progression to CKD was plasma CysC levels. Albuminuria and B2M (directly or as eGFR) were the next best options to be used. The lowest sensitivity was shown by eGFR calculated through creatinine or CysC or even using both parameters; so that, in terms of prognosis, GFR calculation from CysC may not need to be performed. Wider surveys will be needed to confirm our results.

## Conflicts of Interest

The authors declare no conflicts of interest.

## Data Availability

The data that support the findings of this study are available upon request from the corresponding author. The data are not publicly available due to Spanish laws on personal data protection.
